# Research on Correlations of lncRNA ST7-AS1 with Progression and Therapeutic Targets of Esophageal Cancer

**DOI:** 10.5152/tjg.2024.24260

**Published:** 2024-12-16

**Authors:** Xiao Lin, Sijia Sun, JiWen Zhang, Yan Cai, Quan Cheng

**Affiliations:** 1 Department of Gastroenterology, Taizhou Integrated Chinese and Western Medicine Hospital, Wenling, China; 2 Department of Gastroenterology, Huashan Hospital Fudan University, Shanghai, China; 3 Department of Gastroenterology, Shanghai Baoshan Luodian Hospital, Shanghai, China; 4 Department of Traditional Chinese Medicine, Affiliated Hangzhou First People’s Hospital School of Medicine, Westlake University, Hangzhou, China

**Keywords:** ST7-AS1, miR-4262, esophageal cancer, therapeutic biomarker, prognosis

## Abstract

**Background/Aims:**

Esophageal cancer is a highly prevalent gastrointestinal tumor in China, resulting in a significant number of deaths annually. In this paper, we investigated the regulatory role and therapeutic potential of aberrant ST7-AS1 expression in esophageal cancer.

**Materials and Methods:**

The presence of ST7-AS1 in 125 esophageal cancer tissues was identified through RT-qPCR assays. The application of Kaplan-Meier to evaluate survival rates in patients with esophageal cancer. Cell activity was assessed by both CCK-8 and Transwell assays. The luciferase activity assay verified the association of ST7-AS1 with miR-4262.

**Results:**

ST7-AS1 expression in esophageal cancer was noticeably overexpressed compared to the control group. Patients with upregulated ST7-AS1 had shorter survival rates. Silencing ST7-AS1 reduced the proliferation level of esophageal cancer cells, as did the migration and invasion levels. Mechanistically, ST7-AS1 acted as a sponge for miR-4262, affecting the progression of esophageal cancer. This was negatively correlated with ST7-AS1. Moreover, the miR-4262 inhibitor negated the inhibitory effect of silencing ST7-AS1 on cells.

**Conclusion:**

Knockdown of ST7-AS1 may alleviate tumor progression by targeting miR-4262, indicating that ST7-AS1 is anticipated to serve as a therapeutic biomarker for patients with esophageal cancer.

**Main Points**

- ST7-AS1 expression in esophageal cancer was noticeably overexpressed, a trend linked to reduced patient survival rates.
- Silencing ST7-AS1 suppressed cell viability through sponging miR-4262, thereby mediating the development of esophageal cancer.
- ST7-AS1 is considered a prognostic indicator for esophageal cancer.

## Introduction

Esophageal cancer is a highly lethal disease with a high incidence in the digestive system. It ranks sixth among global malignant tumors,^1^ with over 90% of the cases being squamous cell carcinoma and adenocarcinoma.^2^ Despite significant attention and funding dedicated to cancer prevention and treatment in China, the incidence of esophageal cancer remains high. Patients with esophageal cancer often experience difficulty swallowing, a distinct foreign body sensation, sternal pain, or progressive dysphagia.^3^ The standard treatment is surgical resection with preoperative radiotherapy. However, many patients present nonspecific early symptoms, leading to diagnoses in intermediate or advanced stages of the disease. This results in less effective treatment outcomes, poor prognosis, and low quality of life. The advent of molecular targeted therapy over recent decades offers new hope for cancer treatment. In-depth analysis of esophageal cancer pathogenesis and studies on molecular targeted therapy may present a potential treatment method. Hence, identifying specific biomarkers for therapeutic intervention in esophageal cancer patients is a promising avenue to explore.

Long non-coding RNAs (lncRNAs) have intricate secondary or tertiary spatial structures, enabling complex gene regulation.^4^ LncRNAs are linked to the development of various tumors, including esophageal cancer, and are useful for its early detection and prognostic assessment.^5^ LncRNA LUESCC, DGCR5, and LINC00680 have all been shown to mediate the development of esophageal cancer.^6-8^ The dysregulated ST7-AS1, a novel lncRNA, has been associated with tumor progression. For instance, Hu et al pointed out that ST7-AS1 accelerates lung adenocarcinoma metastasis by targeting the miR-181b-5p/KPNA4 axis.^9^ Its oncogenic role has also been emphasized in cervical and gastric cancers.^10^ However, no studies have yet examined ST7-AS1’s role in esophageal cancer. Given the ST7 gene’s widespread expression in human tissues, we hypothesize that a thorough understanding of ST7-AS1’s mechanisms in esophageal cancer could offer promising therapeutic prospects.

This study primarily investigated the levels of ST7-AS1 and its target gene miR-4262 in esophageal cancer tissues and cell samples. Additionally, the potential effect of the abnormal expression of ST7-AS1 on the prognosis and survival of patients was evaluated. The potential mechanisms underlying their regulatory network were further examined, with the goal of providing novel insights for tumor treatment.

## Materials and Methods

### General Information and Inclusion Criteria

This study involved 125 esophageal cancer patients admitted to Shanghai Baoshan Luodian Hospital from August 2015 to February 2017. None of the participants had undergone antitumor therapies like radiation or chemotherapy prior to diagnosis. Patients with a history of coexisting immune diseases or other malignancies were excluded. Esophageal cancer tissue samples, along with some adjacent normal tissue samples (≥ 5 cm), were collected by surgical resection. These were promptly stored in a -80°C refrigerator after being treated with liquid nitrogen. The study was conducted with the consent of the patients and their families and under the accreditation of the Shanghai Baoshan Luodian Hospital ethics committee (approval number: KY2015-02, date: 19 March 2015). All the participants signed the written informed consent.The collected tissue samples were confirmed by the pathologist, and the relevant clinical data were recorded in Table 1.

Patients were monitored for 60 months through telephone communication and outpatient follow-ups. This included an assessment of clinical symptoms, recurrence, and overall prognosis.

### Culture of Experimental Cells

In vitro cell experiments, esophageal epithelial cells HET-1A, and esophageal cancer cells ECA109, OE33, TE6, and EC9706 were purchased from the Cell Bank of Shanghai Academy of Biological Sciences. All cells were maintained in RPMI-1640 medium supplemented with FBS (Gibco, Carlsbad, CA, USA). The incubator conditions were set at 37°C with 5% CO_2_, and the culture medium was changed every 24 hours.

### Transfection

The negative control siRNA (si-NC), silencing ST7-AS1 (si-ST7-AS1), mimic negative control (mimic NC), inhibitor negative control (inhibitor NC), miR-4262 mimic, and miR-4262 inhibitor were produced by GenePharma (Shanghai, China). The primer sequences were (5’-3’): si-NC UCUCCGAACGUGUCACG; si-ST7-AS1 GGGTAACTCAAAAAGCCTG; mimic NC UCACAACCUCCUAGAAAGAG; inhibitor NC CAGUACUUUUGUGUAGUACA; miR-4262 mimic GACAUUCAGACUACCUG; miR-4262 inhibitor AGATTGCTGGGTCACACC.

### RNA Extraction and RT-qPCR

TRIzol reagent (Invitrogen, Carlsbad, CA, USA) was added to tissue samples and esophageal cancer cells for RNA extraction. The RNA was then reverse-transcribed into cDNA. Subsequently, the SYBR Premix EX Taq Kit (TAKARA, Shiga, Otsu, Japan) was applied to real-time quantitative PCR detection. Following amplification, GAPDH and U6 were used as internal parameters for ST7-AS1 and miR-4262. The relative expression was calculated using the 2^-ΔΔCt^ method. The primer sequences were: ST7-AS1 F-5’-ACCCTACTCTGCCTCCCTTA-3’, R-5’-TAGCATCTGCCACCCAA-3’; miR-4262 F-5’-TGCGGGACATTCAGA-3’, R-5’-CCAGTGCAGGGTCCGAGGT-3’; GAPDH F-5’-AGGTCGGTGTGAACGGATT-3’, R-5’-GGGGTCGTTGATGGCAA-3’; U6 F-5’-TGCGGGTGCTCGCTTCGG-3’, R-5’-CCAGTGCAGGGTCCGAGGT-3’.

### In Vitro Cell Assay

ECA109 and EC9706 cells were chosen and cultured in 96-well cell culture plates after being washed with PBS. Following incubation periods of 0, 24, 48, and 72 hours, CCK-8 reagent (Dojindo, Tokyo, Japan) was added to each well. After an additional incubation of 2 hours, the absorbance value was measured at 450 nm using a microplate reader.

The upper chamber of the Transwell was filled with RPMI-1640 medium without serum and inoculated with an esophageal cancer cell suspension, while the lower layer was filled with medium only. After a 24-hour incubation period, the migrated cells were fixed in formaldehyde for 10 minutes and then stained with crystal violet for 20 minutes. The cells were then photographed under a microscope and counted. For the cell invasion assay, Matrigel (BD Biosciences, USA) was spread in the upper chamber of the Transwell. The remaining steps followed the same procedure as the migration assay.

### Luciferase Activity Assay

LncBook 2.0 and LncACTdb 3.0 online databases were used to predict the possible downstream target of ST7-AS1 and draw a Venn diagram. The ST7-AS1 fragment carrying the miR-4262 binding sites was amplified and inserted into the pmirGLO luciferase reporter plasmid (Promega, Madison, WI, USA) to construct ST7-AS1-WT/-MUT. The recombinant plasmids were then combined with either mimic NC or miR-4262 mimic and co-transfected into ECA109 and EC9706 cells. The luciferase activity was measured using the Lipofectamine 2000 reagent.

### Statistical Analysis

The statistical analysis was performed using SPSS 26.0 (SPSS, Chicago, Ill, USA) and GraphPad Prism 9.0 (La Jolla, Calif., USA). Student’s t-test was used for comparing 2 data groups, and ANOVA was used for comparisons among multiple groups. The log-rank method was applied for the statistical test of survival analysis, and Kaplan-Meier was used for survival curve plotting. The correlation between ST7-AS1 and miR-4262 expression was evaluated using the Pearson correlation coefficient. All results were statistically significant with *P *< .05.

## Results

### ST7-AS1 Was Relatively Highly Expressed in Esophageal Cancer

**Figure 1.**
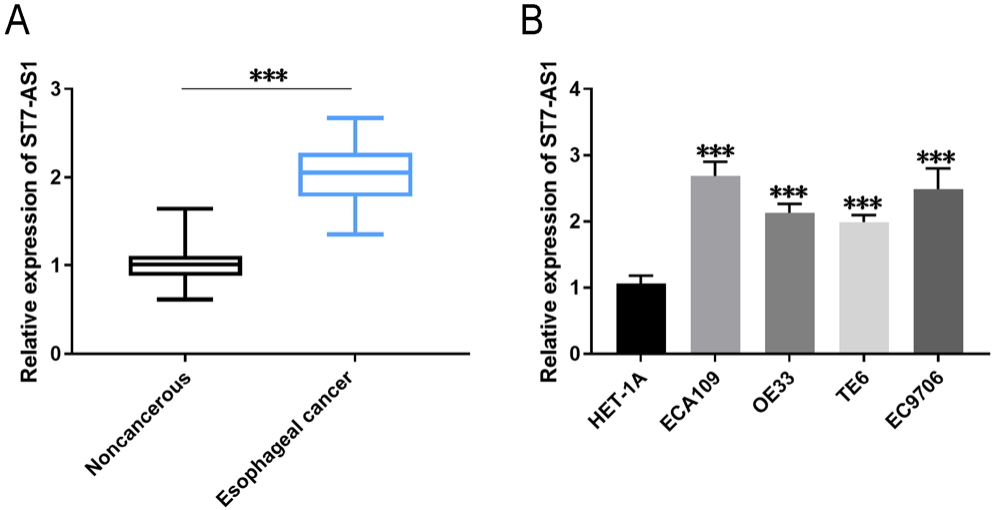
ST7-AS1 expression in esophageal cancer. A: ST7-AS1 level in normal and tissue samples. B: Compared with HET-1A cells, ST7-AS1 was markedly expressed in esophageal cancer cells. ***P < .001.

ST7-AS1 was markedly enhanced in esophageal cancer tissues, as observed using RT-qPCR (Figure 1A). Additionally, ECA109, OE33, TE6, and EC9706 cells exhibited significantly higher expression of ST7-AS1 compared to HET-1A cells (Figure 1B).

In Table 1, the outstanding level of ST7-AS1 was not noticeably correlated with gender, age, tumor size, histological classification, or the smoking and drinking habits of esophageal cancer patients. However, it was influenced by lymph node metastasis and TNM stage.

### Patients with High ST7-AS1 Expression Have Poor Prognosis

**Figure 2.**
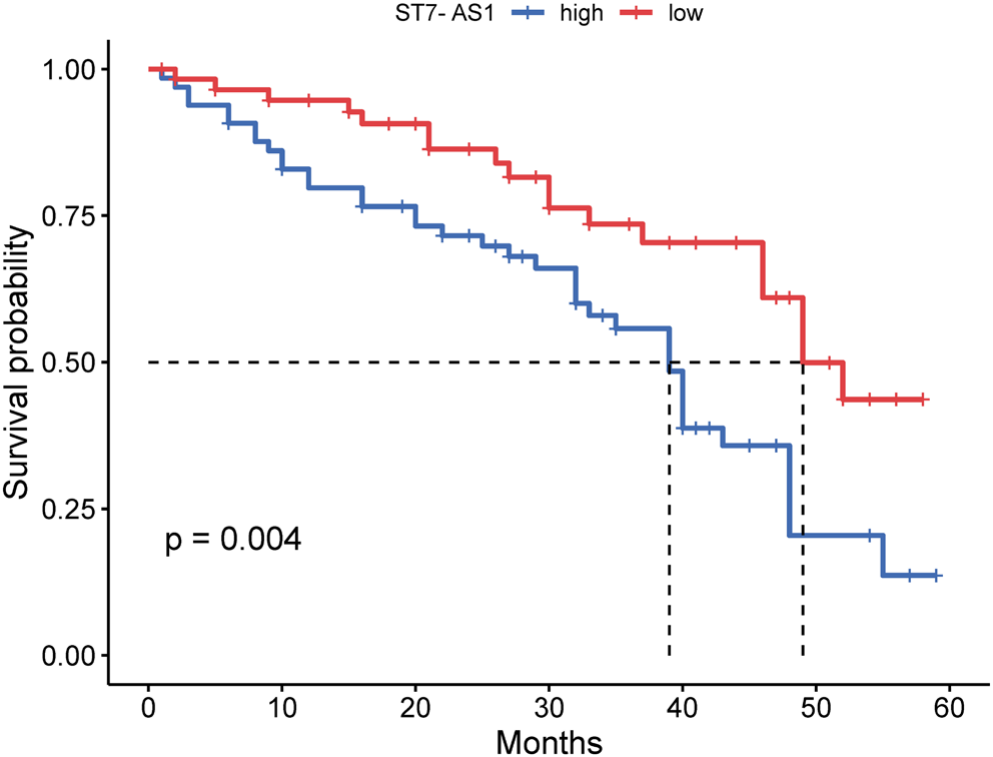
Prominent expression of ST7-AS1 predicts poor prognosis of esophageal cancer patients (*P* = .004).

The Kaplan-Meier method and log-rank test were utilized to analyze the relationship between the relative expression of ST7-AS1 and the prognosis of patients with esophageal cancer. This analysis was conducted through follow-up and summary of esophageal cancer patients. Results are depicted in Figure 2.

The follow-up and summary of esophageal cancer patients revealed a decreased survival rate for those with high-expression ST7-AS1 esophageal cancer. This suggests that high expression of ST7-AS1 predicts a poor prognosis for esophageal cancer patients (*P* = .004, Figure 2).

### Regulation of Cell Viability by Knockdown of ST7-AS1

**Figure 3.**
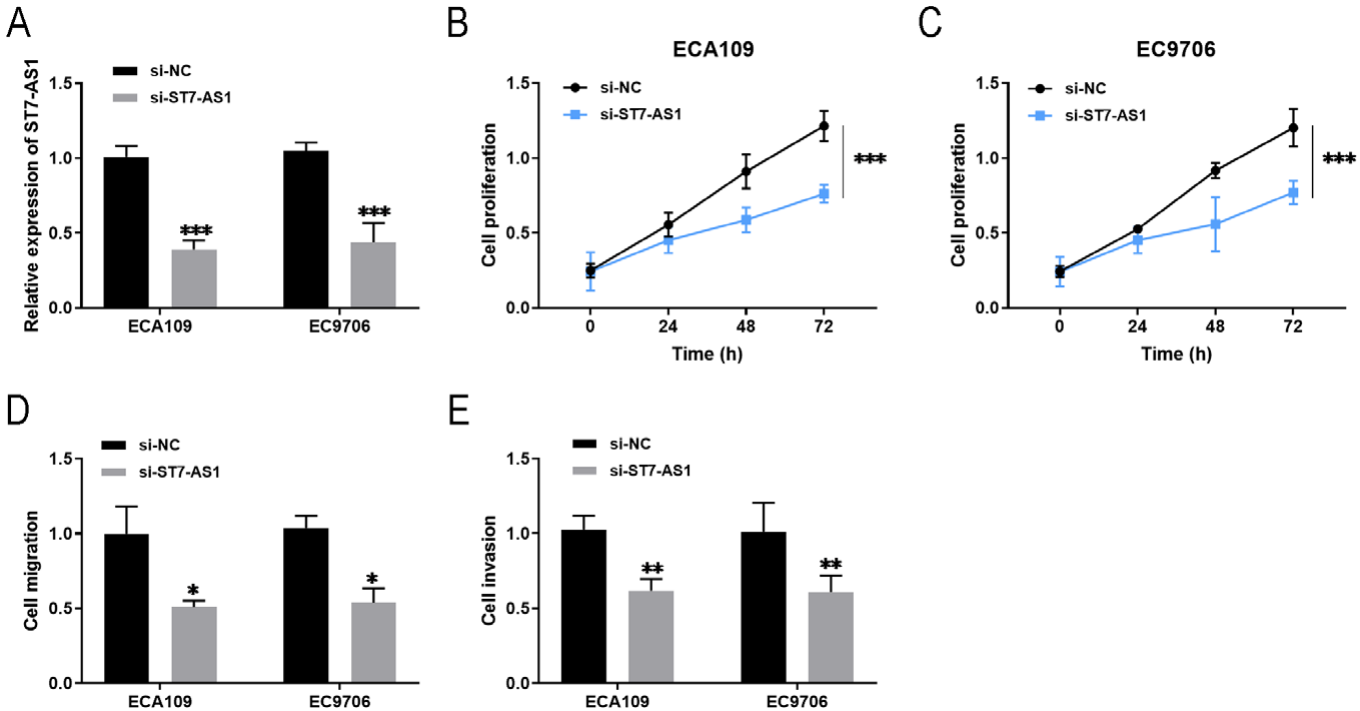
Effects of dysregulated ST7-AS1 on esophageal cancer cell function. A: ST7-AS1 siRNA (si-ST7-AS1) was transfected into ECA109 and EC9706 cells. B-C: ST7-AS1 knockdown suppressed the proliferative capacity of cells. D-E: Effect of silencing ST7-AS1 on migration and invasion of cells. *P < .05, **P < .01, ***P < .001.

In vitro cell experiments resulted in the transfection of si-NC and si-ST7-AS1 into ECA109 and EC9706 cells, respectively. The transfection efficiency is shown in Figure 3A. The proliferation levels of ECA109 and EC9706 cells were down-regulated after transfection with si-ST7-AS1(Figures 3B and 3C). Moreover, the migration number of ECA109 and EC9706 cells decreased after silencing ST7-AS1, as seen in Figure 3D. As illustrated in Figure 3E, the knockdown of ST7-AS1 slowed down the cells’ invasion ability.

### ST7-AS1 Sponge miR-4262 in Esophageal Cancer

**Figure 4.**
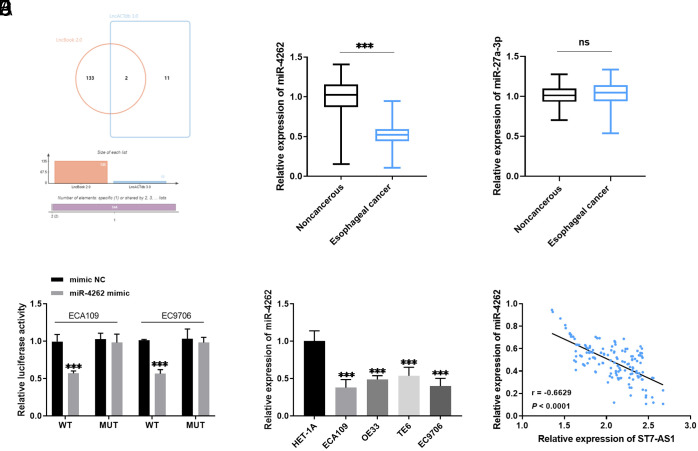
ST7-AS1 sponge miR-4262 regulated the progression of esophageal cancer. A: LncBook 2.0 and LncACTdb 3.0 websites predict downstream targets for ST7-AS1. B-C: Expression of miR-4262 and miR-27a-3p in esophageal cancer tissues. D: Detection of luciferase activity in ECA109 and EC9706 cells. E: miR-4262 was relatively low expressed in esophageal cancer cells. ^ns^*P* > .05; ***P < .001. F: ST7-AS1 level was negatively correlated with miR-4262 (*r* = −0.6629, *P* < .0001).

Two downstream targets of ST7-AS1 were found by Venn diagram verification (Figure 4A). miR-4262 level was downregulated in esophageal cancer tissues (Figure 4B), while the miR-27a-3p level was not significantly changed (Figure 4C). The luciferase activity in the WT group decreased, while there was no significant change in the MUT group, which suggested that ST7-AS1 directly adsorbs miR-4262 and regulates its expression (Figure 4D). miR-4262 expression was also decreased in esophageal cancer cells (Figure 4E). Additionally, there was an inverse correlation between the levels of ST7-AS1 and miR-4262 (Figure 4F; *r* = −0.6629, *P *< .0001).

### Effect of miR-4262 Inhibitor on Cell Viability

**Figure 5.**
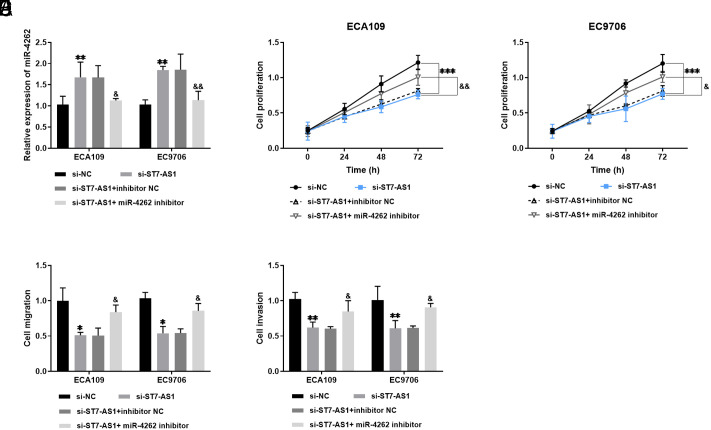
Effect of miR-4262 inhibitor on cell viability. A: miR-4262 was downregulated after transfection with si-ST7-AS1 + miR-4262 inhibitor. B-E: miR-4262 inhibitor restored the inhibitory effect of ST7-AS1 knockdown on the growth and metastasis of esophageal cancer cells. *P < .05, **P < .01, ***P < .001, in comparison with si-NC; &*P* < .05, &&*P* < .01, in comparison with si-ST7-AS1.

In Figure 5A, the relative reduction of miR-4262 levels is shown after the transfection of si-ST7-AS1 + miR-4262 inhibitor in ECA109 and EC9706 cells, compared to the transfection of si-ST7-AS1. The miR-4262 inhibitor counteracts the suppressive effect of si-ST7-AS1 on ECA109 and EC9706 cells (Figure 5B-C). Furthermore, the miR-4262 inhibitor compensates for the inhibitory function of the ST7-AS1 knockdown on cell function (Figure 5D-E).

## Discussion

In China, esophageal cancer incidence and mortality rates are high, with most patients diagnosed at advanced stages. Despite advancements in treatments such as surgery and radiotherapy, their clinical effectiveness often falls short due to the cancer’s high malignancy and patients’ poor prognosis.^11^ The research elaborated that molecular targeted therapy targeting the regulation of related genes to inhibit cancer cell metastasis and proliferation can be effective treatments.^12^ In recent times, the utility of lncRNAs in the context of diseases has become an increasingly focal point of interest. LncRNAs have been proven to have great potential in regulating cell activity, participating in tumor treatment, and affecting the prognosis of patients.^13,14^ There is also evidence that lncRNAs bring new possibilities in the treatment and prognosis of digestive tumors.^15^ For instance, Huang et al proposed that lncRNA PDIA3P1 promotes the progression of esophageal squamous cell carcinoma by modulating OCT4 in a recent report.^16^

ST7-AS1 is an lncRNA located on 7q31.2, and its copy number variation may cause abnormal expression of downstream targeted genes and regulate changes in tumor cell activity.^17^ Existing evidence shows that ST7-AS1 is upregulated in gastric cancer, promoting cell proliferation and inhibiting apoptosis. Conversely, knocking down ST7-AS1 inhibits cell cycle progression.^18^ Furthermore, ST7-AS1 is overexpressed in laryngeal squamous cell carcinoma, propelling carcinogenesis through the CARM1-Sox-2 axis signaling pathway.^19^ In this study, we found that ST7-AS1 is elevated in esophageal cancer, its significantly high expression influenced by lymph node metastasis and TNM staging. Moreover, the Kaplan-Meier curve indicated that increased ST7-AS1 in esophageal cancer could adversely affect patient survival.

In vitro cell assays showed that the growth and viability of esophageal cancer cells decreased after silencing ST7-AS1. This is consistent with previous studies on esophageal cancer, where silencing TUG1 impeded the proliferation and invasion of cancer cells by affecting miR-1294.^20^ Additionally, knockdown of FAM83A-AS1,^21^ ELFN1-AS1,^22^ and SNHG7^23^ reportedly has the potential to inhibit esophageal cancer cell activity. Therefore, it is reasonable to conclude that ST7-AS1 in esophageal cancer can target downstream genes, influencing the tumor process.

LncRNAs interact with miRNAs to function as competing endogenous RNAs, which can abnormally regulate miRNA expression.^24^ Online predictions illustrated a potential interaction between miR-4262 and ST7-AS1, suggesting that ST7-AS1 might directly target miR-4262. This was later confirmed through a luciferase reporter gene assay. As a novel miRNA, miR-4262 was first shown to be associated with cisplatin resistance in adenocarcinoma cells in 2013.^25^ Current evidence indicates that miR-4262 is under-expressed in cervical cancer,^26^ colon cancer,^27^ and osteosarcoma,^28^ which aligns with our experimental results. miR-4262 expression was reduced in esophageal cancer, and there was a negative correlation between ST7-AS1 and miR-4262. Moreover, research by Liu and colleagues demonstrated that miR-4262 is expressed at lower levels in esophageal cancer and slows tumor progression by negatively controlling the downstream gene KLF6.^29^ The inhibitory effect of silencing ST7-AS1 on cells was counteracted by a miR-4262 inhibitor.

Based on these findings, the ST7-AS1 sponge of miR-4262 appears to play a negative regulatory role, thereby participating in the biological functions of esophageal cancer cells and tumorigenesis. Unfortunately, the number of patients included in this study was limited, and there was a lack of discussion regarding the correlation between ST7-AS1 expression and factors such as the clinical setting. This requires further attention in subsequent work.

Generally, ST7-AS1 expression was elevated in esophageal cancer. Silencing ST7-AS1 suppressed cell viability by acting as a sponge for miR-4262, which subsequently influenced the development of esophageal cancer. ST7-AS1 may serve as a potential therapeutic target for future clinical treatments.

## Availability of Data and Materials:

The data that support the findings of this study are available on request from the corresponding author.

## Figures and Tables

**Table 1:** The Relationship Between lncRNA ST7-AS1 and the Clinical Features of Esophageal Cancer Patients

Features	lncRNA ST7-AS1 Expression (N = 125)		*P*
	High (n = 65)	Low (n = 60)	
Gender			.763
Male	34	33	
Female	31	27	
Age			.873
≤ 60 years	37	35	
> 60 years	28	25	
Tumor size			.086
≤ 3 cm	37	43	
> 3 cm	28	17	
Histological classification			.135
Squamous cell carcinoma	52	41	
Adenocarcinoma	13	19	
Alcohol consumption			.465
No	41	34	
Yes	24	26	
Smoking status			.423
No	30	32	
Yes	35	28	
Lymph node metastasis			.041
Negative	40	47	
Positive	25	13	
TNM stage			.028
I-II	33	42	
III-IV	32	18	
