# Erratum

**DOI:** 10.5152/tjg.2025.030626

**Published:** 2025-02-28

In the original article authored by Lin et al, titled “Research on Correlations of lncRNA ST7-AS1 with Progression and Therapeutic Targets of Esophageal Cancer” (Turk J Gastroenterol. 2025; 36(2): 82-88. DOI: 10.5152/tjg.2024.24260) published in the February 2025 issue of Turkish Journal of Gastroenterology, the statement that the first two authors contributed equally to the article has not been included and the corresponding author information was written incorrectly by an oversight. Subsequently, the correct author contributions added and the corresponding author information is corrected, and the online version of the original article has been updated accordingly. You can access the revised version of this original article via the following link: https://www.turkjgastroenterol.org/en/research-on-correlations-of-lncrna-st7-as1-with-progression-and-therapeutic-targets-of-esophageal-cancer-137302

