# Erratum

**DOI:** 10.5152/ArchRheumatol.2025.00001

**Published:** 2025-12-01

In the original article authored by Çınar E, Türkoğlu Aytar MB, Baklacı M, Hepgüler S, Barutçuoğlu B., titled “Fecal calprotectin levels in patients with fibromyalgia: A cross-sectional study” (ARCH RHEUMATOL. 2025, 4, 39, doi: 10.46497/ArchRheumatol.2024.10557.) published in the fourth issue of Archives of Rheumatology, the funding statement was declared incorrectly by an oversight. Subsequently, the funding statement was corrected, and the online version of the original article has been updated accordingly. You can access the revised version of this original article via the following link: https://archivesofrheumatology.org/index.php/pub/article/view/1444

